# Element Concentrations in Muscle and Liver Tissue of Two Eel Species from the Incomati River, Mozambique

**DOI:** 10.1007/s00128-023-03795-5

**Published:** 2023-09-10

**Authors:** Johannes H. Erasmus, Shaun Herselman, Victor Wepener

**Affiliations:** Water Research Group, Unit for Environmental Sciences and Management, North-West University, 11 Hoffman St, Potchefstroom, 2520 South Africa

**Keywords:** *Anguilla mossambica*, *Anguilla marmorata*, Bioaccumulation, Chronic human health risks

## Abstract

Many rural communities in Mozambique get their income from fisheries, as well as consume fish as an inexpensive source of protein. In Mozambique, fish consumption from small-scale fisheries dependent families can range between 60 and 150 kg/person/year. Anguillid eels have important social value to vulnerable local communities in the Incomati Basin. Anguillid eels in the lower Incomati Basin are targeted for consumption, as well as cultural rituals by traditional healers. The aim of the present study was to determine trace element concentrations in environmental matrices, bioaccumulation patterns in muscle and liver tissue of two *Anguilla* species (*Anguilla mossambica* and *Anguilla marmorata*), as well as the chronic health risk these elements pose to regular consumers in the lower Incomati River. Bioaccumulation of elements from the water had a higher effect on both muscle and liver tissues, where only the bioaccumulation of Pb in the sediments played a major role. From the human health risk assessment, Hg pose non-carcinogenic risk, whereas As, Cr, and Ni pose carcinogenic risks.

**Supplementary Information:**

The online version contains supplementary material available at 10.1007/s00128-023-03795-5.

## Introduction

Fish is an inexpensive source of protein and are thus harvested by low-income communities in developing countries. In Mozambique, it is estimated that families who are dependent on small-scale fisheries can consume between 60 and 150 kg of fish per person per year (WIOMSA, 2021). These fish are harvested by subsistence fishers from local rivers, which could pose health risks to humans that regularly consume them (Erasmus et al. 2022). These health risks are influenced by the water body from which the fish are harvested. Fish from contaminated rivers could have adverse effects on the individuals that consume them (Addo-Bediako et al. 2014). Fish contaminated with metals could cause non-carcinogenic and carcinogenic risks (Mannzhi et al. 2021). Trace elements bioaccumulate in the tissues of aquatic organisms, such as fish, through food, water, as well as sediments (Lebepe et al. 2020). Most elements do not biomagnify, except for Hg, but rather bioaccumulate in lower feeding groups, thus organisms at a lower trophic level may accumulate more trace elements than those at a higher trophic level (Erasmus et al. 2022).

Anguillids such as the African longfin eel (*Anguilla mossambica*) and the giant mottled eel (*Anguilla marmorata*) are both western Indian Ocean species (Hanzen et al. 2022). Both of these species are under threat due to human structures that hinder their migration patterns, as well as overfishing, especially in Mozambique (WIOMSA, 2021). The African longfin eel is a carnivorous species of which the adults feed on fish, frogs, and benthic macroinvertebrates such as crabs (Skelton 2001; Parker 2010). *Anguilla marmorata* is also a carnivorous species and has the same feeding habits as *A. mossambica* (Skelton 2001; Huyen and Linh 2019), which is widely distributed and can be found in the western Indian Ocean, as well as in the North and South Pacific Oceans (Minegishi et al. 2008). These eel species have great social value to local communities in the lower Incomati River catchment and are targeted for consumption, as well as several cultural rituals by traditional healers (WIOMSA, 2021). Anguillid eels in the lower Incomati River catchment also provide income to several local fishers, as these eels are illegally exported to Asia as a delicacy (WIOMSA, 2021). According to Luo et al. (2013), the consumption of *A. marmorata* is a longstanding tradition in southeast China.

The present study therefore hypothesised that (1) due to the same feeding habits and exposure to the same environment, both these species element bioaccumulation from the environment will be similar, and (2) these eels are not exposed to a highly anthropogenic impacted area, thus will not pose any human health risks to consumers. This study therefore assessed the non-carcinogenic and carcinogenic human health risks associated with regular consumption of these two *Anguilla* species, as they are not only consumed by local communities but also on an international scale. This is the first report of element accumulation from these two eel species in Africa.

## Materials and Methods

### Study Area

**Fig. 1:**
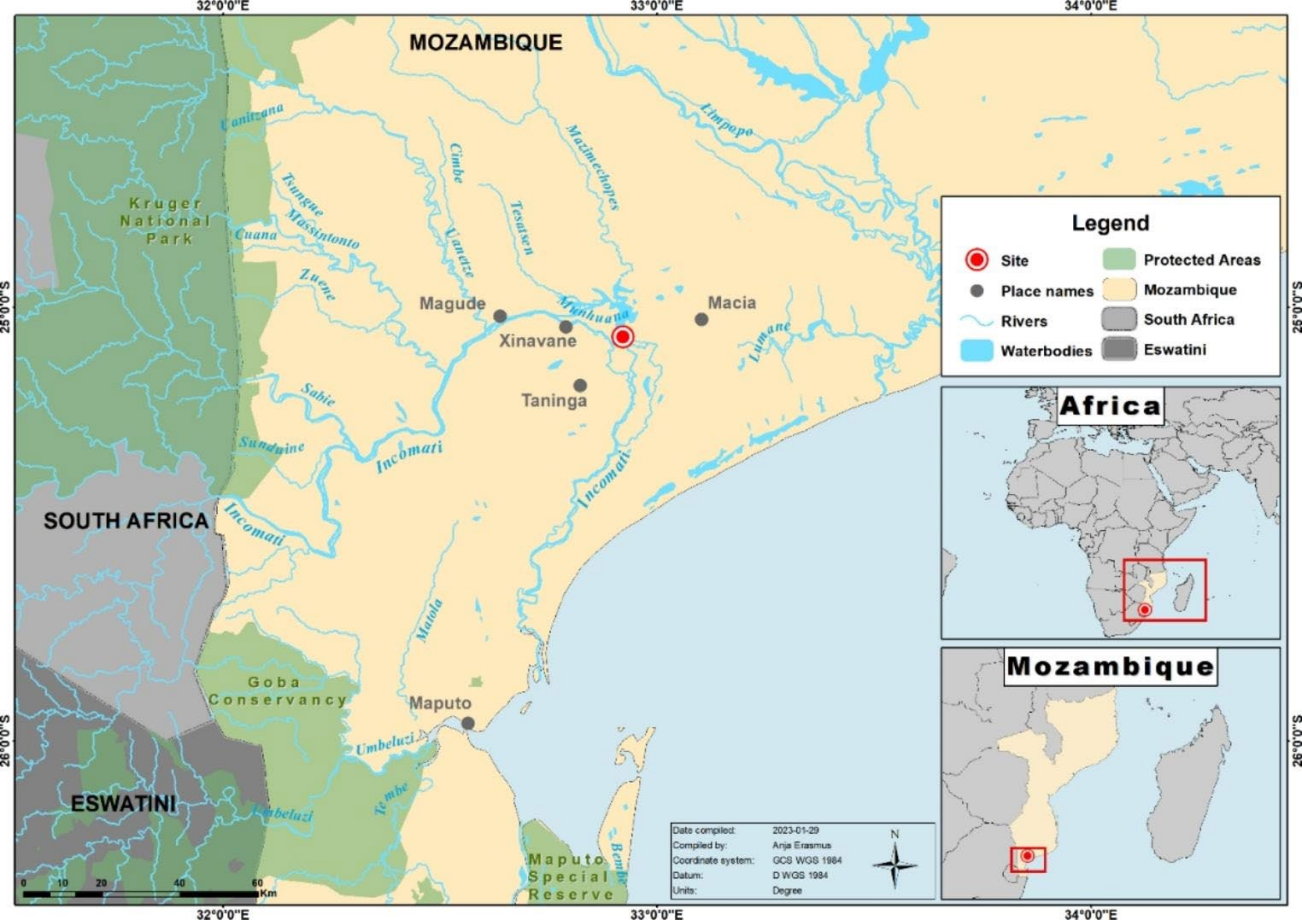
Map of the study area, indicating the sampling site (red circle), Incomati River Basin, the major tributaries of the Incomati River, as well as neighbouring countries (Eswatini, South Africa)

The Incomati River catchment is a semi-arid transboundary catchment that drains through South Africa, Eswatini, Mozambique and into the Indian Ocean. Most of the catchment is located within the border of South Africa (63%), 32% in Mozambique and only 5% in Eswatini (Kleynhans et al. 2013). The catchment has multiple competing water demands and drains areas with intensive agricultural and forestry activities, urban developments and coal combustion power stations, as well as coal and gold mining activities within South Africa (Slinger et al. 2010; Saraiva Okello et al. 2015). In Mozambique, the main water use is irrigated agriculture such as sugarcane, that occupies approximately 23,000 ha (Gallego-Ayala and Juízo 2014). The sampling site in the present study was located in the Incomati River near Xinavane in the Maputo district, Mozambique (Fig. 1). This site was selected as it is in the main region where eels are actively harvested for local and international export consumption purposes.

### Field Sampling

Water, sediment, and eel samples were collected in the Incomati River near Xinavane during June 2022. The necessary permit and ethics were obtained prior to sampling (C2021/2022 − 00512) (AS/UMP/20,220,115). In situ water quality parameters (pH, electrical conductivity (EC), temperature, dissolved oxygen (DO) were measured at the site with handheld meters (ExStik II EC500 and ExStik II DO600, Extech Instruments). Water samples were collected in pre-cleaned polypropylene containers and frozen until analysis could be done. Sediment samples were collected in pre-cleaned polypropylene containers and frozen to prevent organic decomposition (Wepener et al. 2011). Seven *Anguilla mossambica* and 12 *Anguilla marmorata* individuals were caught using fyke nets and euthanised by severing the spinal cord. Total length (TL) and mass were recorded and subsequently, muscle and liver tissue were extracted and stored in pre-cleaned polypropylene tubes and frozen until further analysis (Wepener et al. 2011). The TL ranged between 510 and 790 mm, as well as 710 and 1 250 mm with a mean of 616 ± 99 mm and 875 ± 169 mm, while the mass ranged between 290 and 970 g, as well as 710 and 3 900 g with a mean of 490 ± 240 g and 1 463 ± 1 003 g for *A. mossambica* and *A. marmorata* individuals, respectively.

### Laboratory Analyses

Water samples were defrosted and analysed at room temperature for variables such as ammonium, nitrates, nitrites (sum of ammonium, nitrates and nitrites form total nitrogen concentration), chloride, ortho-phosphate, sulphate and total alkalinity using a Spectroquant Pharo 300 spectrophotometer, where after the samples were prepared for element analysis by filtering it through a cellulose nitrate filter (0.45 μm, Sartorius Stedim Biotech) and acidified with HNO_3_ (65% supra pure quality, Merck) to an acid concentration of 1‰ (Erasmus et al. 2020a). Sediment samples were freeze-dried (FreeZone® 6, Labconco) and subsequently, the particle size was determined using methods described in Wepener and Vermeulen (2005). Particle size categories that were applied include: gravel (> 4000 μm), very coarse sand (4000 − 2000 μm), coarse sand (2000 − 500 μm), medium sand (500 − 212 μm), fine sand (212 − 65 μm) and mud (< 65 μm) (Erasmus et al. 2020a). Subsequently, approximately 0.2 g (dry weight) of the sediment was digested in a mixture of 7.5 ml HCl (37%, supra pure quality, Merck) and 2.5 ml HNO_3_ (65%, supra pure quality, Merck) (Erasmus et al. 2020b). The muscle and liver samples of the eel species were prepared for metal analysis by freeze-drying (FreeZone® 6, Labconco) the samples, after which approximately 0.2 g (dry weight) of each sample was digested in a mixture of 7.5 ml HNO_3_ (65%, supra pure quality, Merck) and 2.5 ml HCl (37%, supra pure quality, Merck) (Erasmus et al. 2020b). Digestions were done using an advanced microwave digestion system (Ethos Easy, Milestone), with 100 ml TFM® vessels (Milestone). After the sediment and eel samples were digested it was transferred into a 50 ml volumetric flask and diluted with MilliQ® water (Erasmus et al. 2020a). Samples were then analysed for elements (As, Cd, Cu, Ni, Pb and Zn) using quadrupole inductively coupled plasma mass spectroscopy (ICP-MS) (PerkinElmer, Elan 6000) equipped with an auto sampler system (PerkinElmer, AS-90), while concentrations of Cr were determined on an atomic absorption spectrometer (AAS) (PerkinElmer, AAnalyst 600) equipped with Zeeman-effect background correction. Concentrations of Hg were analysed on a flow injection mercury system (FIMS) (PerkinElmer, FIMS 400). Quality control of the element analyses were performed using reference materials for sediment (NCS CD 73,310; stream sediment certified reference material, China National Analysis Centre for Iron and Steel) and biota (ERM – CE 278k; mussel tissue, Institute for Reference Materials and Measurements, European Commission) for all elements of interest. Recovery rates for all elements were recorded within a 20% deviation of the certified concentrations (Table 1).

**Table 1:** Limit of detection (LOD), limit of quantification (LOQ) reported in mg/kg dry weight (DW) in sediment and eel tissue samples, as well as recovery rates (%) of the elements of interest from different certified reference materials

Element	Sediment		Eel muscle		Eel liver		% Recovery	
	LOD	LOQ	LOD	LOQ	LOD	LOQ	NCS DC 73,310	ERM – CE 278k
As	0.017	0.052	0.011	0.034	0.022	0.067	117	87
Cd	0.0002	0.0006	0.0011	0.0033	0.0022	0.0065	91	104
Cr	0.011	0.032	0.034	0.103	0.012	0.035	87	82
Cu	0.003	0.008	0.054	0.162	0.106	0.318	91	116
Hg	0.0009	0.0027	0.0042	0.0126	0.0018	0.0053	110	98
Ni	0.053	0.159	0.034	0.103	0.067	0.202	84	118
Pb	0.035	0.110	0.040	0.121	0.079	0.238	81	102
Zn	0.063	0.190	0.064	0.193	0.013	0.038	91	85

### Statistical Analyses

Statistical significance was determined at p < 0.05 for the variation between element concentrations in muscle and liver tissues within an eel species, as well as between the two eel species. Following the tests for normality and homogeneity, 2-way ANOVA and Tukey’s multiple comparison test, and Sidak multiple comparison tests were performed using GraphPad Prism v9. Principal component analyses (PCAs) were constructed to assess the bioaccumulation patterns in the muscle and liver tissue of the two eel species with water and sediment concentrations overlayed as supplementary variables. All the data used in the PCAs were log transformed: log (x + 1). Bioconcentration factors (BCF = C_eel_/C_water_) and biota-sediment accumulation factors (BSAF = C_eel_/C_sediment_) were calculated as the ratio of the average trace element concentration in eel muscle tissue for each of the species, per quantified element concentration of the filtered (0.45 μm) water and sediment, respectively. This was done to evaluate the trace element transfer from water and sediment to the eel tissues. An assessment based on the human health risks associated with the consumption of *A. mossambica* and *A. marmorata* was done using equations described by Erasmus et al. (2022) to calculate the non-carcinogenic, as well as carcinogenic health risks of the different elements.

## Results and Discussion

### Water and Sediment

The dominant drivers in water quality variables at the Xinavane site were total alkalinity, high pH value and high electrical conductivity, while dissolved element concentrations were in the order As > Zn > Ni > Cr > Cd > Hg (Supplementary Data, Table S1). The fine sand particle size was dominant at the site, comprising of 75% of the particle size composition, while element concentrations in the sediment were in the order Cr > Ni > Zn > Cu > Pb > As > Cd > Hg (Supplementary Data, Table S1).

### Element Concentrations in *Anguilla mossambica* and *Anguilla marmorata*

**Fig. 2:**
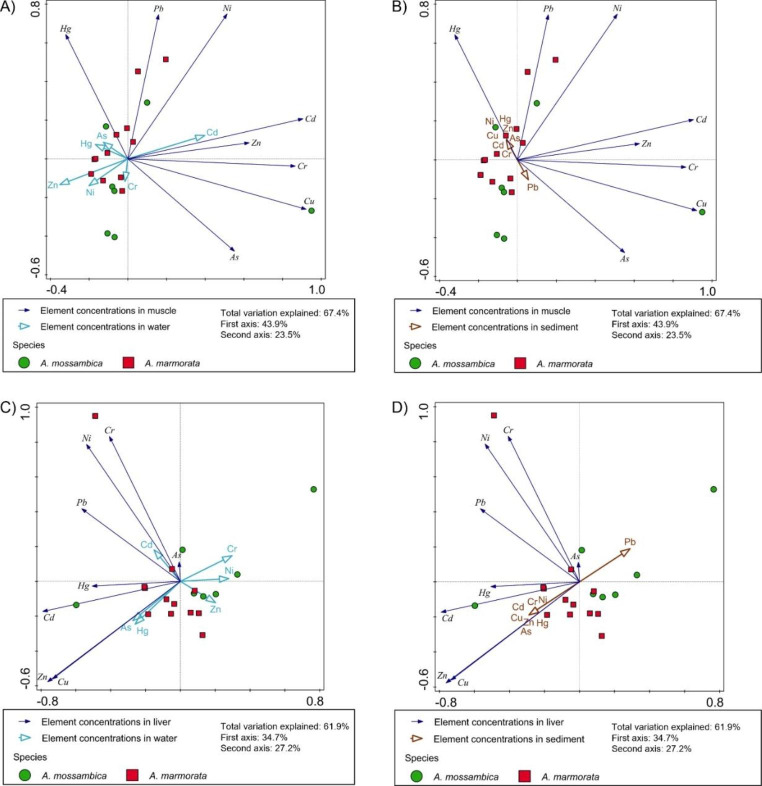
PCA biplots with element concentrations in muscle tissue with element concentrations in water (**A**) and sediment (**B**) overlayed as supplementary data, as well as element concentrations in liver tissue with element concentrations in water (**C**) and sediment (**D**) overlayed as supplementary data

Significant differences between the two eel species, as well as between the muscle and liver tissue within the species were evident (Supplementary data, Figure S1). Concentrations of As, Cd, and Zn were significantly higher in the liver tissues compared to the muscle tissues of both eel species. Although not significant, the trend in concentrations of As, Cd and Cu in muscle tissues, as well as concentrations of Cr and Hg in the liver of *A. mossambica* were generally higher compared to *A. marmorata*, while concentrations of As in the liver were significantly higher. On the other hand, concentrations of Cd, Cu, Ni, and Zn in liver tissue, and Pb in both tissues of *A. marmorata* were higher compared to *A. mossambica*, with only Cd in the liver significantly higher. To the authors knowledge, the present study is the first to report bioaccumulated element concentrations in *A. mossambica* in Africa, as well as in its distribution range, while several studies conducted in various rivers in Vietnam, New Caledonia, and Indonesia, reported element concentrations in *A. marmorata* (Le et al. 2009, 2010, 2012; Sarong et al. 2013; Germande et al. 2022). Comparing these element concentrations with concentrations found in *A. marmorata* from four rivers in the central part of Vietnam, where pesticides and fertilizers have been used intensively for agriculture (Le et al. 2009), the concentrations for Cd, Cr, and Zn in the Incomati River were within the same range, whereas Cu concentrations were considerably lower and concentrations of Pb were higher in the present study. Mercury concentrations were several folds lower in the muscle and liver tissue, whereas Cd, as well as Cr concentrations were in the same range. Copper, as well as Zn concentrations were considerably higher than reported in muscle tissue of *A. marmorata* from the Ba River, Vietnam (Le et al. 2010). The concentrations for Cu and Zn in the liver tissue of *A. marmorata* were within the same range, whereas concentrations of Cd and Hg were higher and Cr and Pb were lower in the Incomati River compared to the element concentrations in the Ba River, Vietnam (Le et al. 2012). Cadmium concentrations were within the same range as reported from *A. marmorata* in the Keureto River, Indonesia (Sarong et al. 2013). Nickel concentrations in the muscle tissue of *A. marmorata* was very low compared to the liver (Germande et al. 2022), the same is true for muscle and liver tissue collected from *A. marmorata* in the Incomati River. The element bioaccumulation patterns in the muscle tissue (Fig. 2A) of the two eel species were very similar and based on the BAFs the main exposure route was through water (Table 2). However, clear species differences in bioaccumulation patterns were observed in the liver tissues (Fig. 2C and D) with higher element concentrations (and BAFs) being recorded in *A. marmorata* compared to *A. mossambica* (Table 2). Like muscle tissue, the liver BAFs indicated that water was the main route of exposure with sediments contributing only to Hg, Cu and Zn bioaccumulation. The differences that were found in element bioaccumulation in the livers of the two species may be attributed to species specific metal metabolism strategies. This is not uncommon as Weber et al. (2020) found that differential hepatic metallothionein expression contributed to significant differences in metal bioaccumulation in liver of fish species from the same metal contaminated site.

**Table 2:** Bioconcentration factors (BCF) calculated as the ratio of the trace element concentrations in eels (*Anguilla mossambica*, and *Anguilla marmorata*) and the trace element concentrations in water, as well as biota-sediment accumulation factors (BSAF) calculated as the ratio of the trace element concentrations in eels and trace element concentration in sediment. Dissolved concentrations of Cu and Pb in water, as well as concentrations of Cu in muscle tissue of *A. marmorata* were below the detection limits

Elements	Bioconcentration factors (BCF)			
	***A. mossambica***		***A. marmorata***	
	Muscle	Liver	Muscle	Liver
As	180	394	105	190
Cd	400	1 760	220	3 000
Cr	244	380	231	317
Cu	-	-	-	-
Hg	4 027	4 135	4 103	3 318
Ni	185	242	200	457
Pb	-	-	-	-
Zn	46 000	138 000	48 000	173 000
	**Biota-sediment accumulation factors (BSAF)**			
As	0.12	0.23	0.072	0.10
Cd	0.31	1.5	0.17	2.5
Cr	0.007	0.011	0.007	0.009
Cu	3.1	4.6	-	5.5
Hg	35	36	35	29
Ni	0.0035	0.0050	0.0037	0.0090
Pb	0.017	0.020	0.021	0.020
Zn	1.3	3.8	1.8	4.8

### Human Health Risks

Fish are rich sources of proteins and nutrients and are low in fatty acids, which makes it ideal for human consumption (Mannzhi et al. 2021). Subsistence fishers rely on fish harvested from local lakes, rivers, and impoundments for income and a source of protein (Addo-Bediako et al. 2014; Luo et al. 2013). Local communities near the Incomati River catchment are subsistence farmers and rely on fish from the river as an easily accessible source of protein. It is evident from the bioaccumulation results, that there are elements that accumulated in the muscle and liver tissue of *A. mossambica* and *A. marmorata*. There were no observable eel health impairments in the present study, but this does not mean that these element concentrations do not pose any health risks to regular fish consumers. The conservative human health risks calculated were based on adults with a mass of 60 kg, since there isn’t any exact information available on the fish consumption by local communities in the Incomati River catchment. The assessment of the human health risk was not considered for individuals that are at a higher risk due to chronic exposure to trace elements, such as pregnant woman, lactating mothers, infants, and children (Erasmus et al. 2022).

#### Non-carcinogenic Health Risks

For non-carcinogenic risks, if the hazard quotient (HQ) is higher than one, it indicates that there is a high probability of adverse health effects. According to the human health risk assessment, the concentrations of Hg within these eel species were the only element with HQ values higher than one and are considered not acceptable for safe human consumption (Table 3). Although only the concentrations of Hg exceeded the safe human consumption levels for non-carcinogenic risks, it is important to keep in mind that a mixture of different trace elements occur within these eel species and could potentially pose an even greater risk to people who consume these eels. Other pollutants in the aquatic system (e.g., organic compounds) may also increase the health risk associated with eel consumption. According to Teunen et al. (2022), mercury can act as a potent neurotoxicant, especially in its organic, methylated form (i.e., methylmercury – MeHg), and will interfere both with perceptive systems (i.e., vision, hearing) and movements (i.e., immobility, uncontrollable movements) for humans, even at low concentrations.

**Table 3:** The mean and standard deviation of hazard quotients (HQs), cancer risk (CR), as well as maximum safe consumption limit per day for the two eel species *Anguilla mossambica*, and *Anguilla marmorata* from the Incomati River, Mozambique, calculated on the average trace element concentration in the muscle tissue, supposing a person of 60 kg consumes one fish meal (150 g), twice a week for the HQ or daily for CR. Hazard quotients and CR values for most of the investigated trace elements were far below zero and only values of HQ > 1, indicating a high probability of adverse health effects, as well as CR > 10^− 4^, indicating an unacceptable risk to humans that consume these fish, are included in this table

Elements	*A. mossambica*	*A. marmorata*
Hazard quotient for non-carcinogenic risk (HQ)		
Hg	2.5 ± 0.55	2.4 ± 0.26
Cancer risk (CR) (10^− 4^)		
As	5.6 ± 1.8	3.2 ± 0.97
Cr	3.0 ± 1.3	2.0 ± 1.0
Ni	1.1 ± 0.66	1.2 ± 1.1
Maximum safe consumption limit per day (g)		
As	701 ± 383	1 148 ± 311
Cr	157 ± 83	217 ± 121
Hg	26 ± 5.8	23 ± 3.1
Ni	1 263 ± 637	1 012 ± 293

#### Carcinogenic Risk

A public screening criterion for carcinogens is normally set at a carcinogenic risk level of 10^− 6^, if the cancer risk (CR) is below 10^− 6^ it can be considered as an acceptable risk. When the CR is between 10^− 6^ and 10^− 4^ there is a level of concern, while a CR above 10^− 4^ is considered as unacceptable (USEPA, 2005). The carcinogenic risk assessment for elements in the two eel species indicated that As, Cr, and Ni had a higher carcinogenic risk than other elements and its CR ranged between 5.6 × 10^− 4^ and 3.2 × 10^− 4^ for As, 3.0 × 10^− 4^ and 2.0 × 10^− 4^ for Cr, and 1.1 × 10^− 4^ and 1.2 × 10^− 4^ for Ni for *A. mossambica* and *A. marmorata*, respectively (Table 3). This estimates that 6 and 3 out of 10 000 people have the chance of developing cancer from As due to consumption of *A. mossambica* and *A. marmorata*, respectively from the Incomati River. When compared to the target risk of > 1 × 10^− 4^, the CR for As, Cr, and Ni are considered unacceptable and should be a concern to people who consume these eels. As indicated by Erasmus et al. (2022), As is known to be associated with several different cancers (skin, bladder, and lungs), while ingestion of Cr can cause stomach, liver, and kidney cancers. According to Cameron et al. (2011), water soluble nickel compounds by themselves are not considered complete human carcinogens due to the predicted lack of bioavailability of nickel ion at target sites. However, Ni can inhibit the repair of damaged DNA even at low concentrations (Erasmus et al. 2022).

#### Maximum Safe Consumption Limits

Maximum safe consumption to prevent non-carcinogenic health risks associated with Hg contamination was 26 and 23 g for *A. mossambica* and *A. marmorata* fillet from the Incomati River per day, respectively. Even though these small portions of fish are safe to consume, it is not realistic or sufficient to sustain people who rely on fish as an inexpensive and easily accessible source of protein. Maximum consumption limits to prevent carcinogenic health risks ranged between 701 and 1 148 g fillet for As, between 157 and 217 g fillet for Cr and between 1 263 and 1 012 g fillet for Ni of *A. mossambica* and *A. marmorata*, respectively. These portions are more sustainable as protein source; however, this was only based on a single element calculation, while these eel tissues are contaminated with a mixture of elements and other pollutants and may therefore pose an even greater risk. With the maximum safe consumption limit being very low, ≤ 21 g per day based on the non-carcinogenic risk of Hg, it should be an indication that even if small portions are consumed there is still an increased risk to human health.

## Conclusion

The present study assessed the element concentrations in environmental matrices (water and sediment) and bioaccumulation of these elements by two eel species from their environment in both muscle and liver tissue, as well as the human risks associated with trace element concentration through the consumption of these eel species. The data do not support the two hypotheses as there was a distinct difference in the metal accumulation in the livers of the two eel species. This was attributed to species specific metal metabolism strategies. Even though there are no known anthropogenic sources of metals in the vicinity, the human health risk assessment did reveal that Hg in muscle of both eel species pose non-carcinogenic risks, while concentrations of As, Cr, and Ni pose carcinogenic health risks.

### Electronic supplementary material

Below is the link to the electronic supplementary material.

Supplementary Material 1
