# Supplementary material for: Element Concentrations in Muscle and Liver Tissue of Two Eel Species from the Incomati River, Mozambique
Source: Bull Environ Contam Toxicol. 2023 Sep 10;111(3):34. doi: 10.1007/s00128-023-03795-5 (PMC10493202; doi:10.1007/s00128-023-03795-5)
Supplement: Supplementary file 1 — Supplementary Material 1 [file 128_2023_3795_MOESM1_ESM.docx]

Element concentrations in muscle and liver tissue of two eel species from the Incomati River, Mozambique

Johannes H. Erasmus^*^, Shaun Herselman, Victor Wepener

Water Research Group, Unit for Environmental Sciences and Management, North-West University, 11 Hoffman St, Potchefstroom, 2520, South Africa

**^*^** Corresponding author: J.H. Erasmus (hannes.erasmus@nwu.ac.za)

ORCID ID:

J.H. Erasmus 0000-0001-9056-5424

S. Herselman 0000-0002-5946-3021

V. Wepener 0000-0002-9374-7191

Email addresses:

J.H. Erasmus hannes.erasmus@nwu.ac.za

S. Herselman herselmanshaun@gmail.com

V. Wepener victor.wepener@nwu.ac.za

**Supplementary data**

Table S1: Mean with standard deviation of the water and sediment quality variables measured in the Incomati River, Mozambique. Dissolved Cu and Pb concentrations in water were not detected (ND).

| Water quality variables | |
| --- | --- |
| pH | 8.56 ± 0.14 |
| Electrical conductivity (µS/cm) | 1 162 ± 32.3 |
| Temperature (°C) | 21.7 ± 0.32 |
| Dissolved oxygen (mg/l) | 9.7 ± 0.15 |
| Total Nitrogen (mg/l) | 0.34 ± 0.006 |
| Chloride (mg/l) | 0.54 ± 0.007 |
| Ortho-phosphate (mg/l) | 0.15 ± 0.08 |
| Sulphate (mg/l) | 26 ± 1.0 |
| Total alkalinity (mg/l CaCO_3_) | 109 ± 2.9 |
| As (µg/l) | 1.7 ± 0.14 |
| Cd (µg/l) | 0.051 ± 0.009 |
| Cr (µg/l) | 2.1 ± 0.12 |
| Cu (µg/l) | ND |
| Hg (µg/l) | 0.18 ± 0.018 |
| Ni (µg/l) | 0.73 ± 0.07 |
| Pb (µg/l) | ND |
| Zn (µg/l) | 1.3 ± 0.22 |
| Sediment quality variables | |
| Gravel (%) | 0.58 ± 0.84 |
| Very coarse sand (%) | 2.7 ± 3.5 |
| Coarse sand (%) | 5.7 ± 6.8 |
| Medium sand (%) | 22.7 ± 18.8 |
| Fine sand (%) | 74.7 ± 49.5 |
| Mud (%) | 1.7 ± 1.6 |
| Organic content (%) | 1.1 ± 0.62 |
| As (mg/kg DW) | 2.9 ± 0.92 |
| Cd (mg/kg DW) | 0.064 ± 0.02 |
| Cr (mg/kg DW) | 73 ± 4.2 |
| Cu (mg/kg DW) | 17 ± 9.4 |
| Hg (mg/kg DW) | 0.021 ± 0.002 |
| Ni (mg/kg DW) | 37 ± 33 |
| Pb (mg/kg DW) | 27 ± 21 |
| Zn (mg/kg DW) | 36 ± 3.5 |


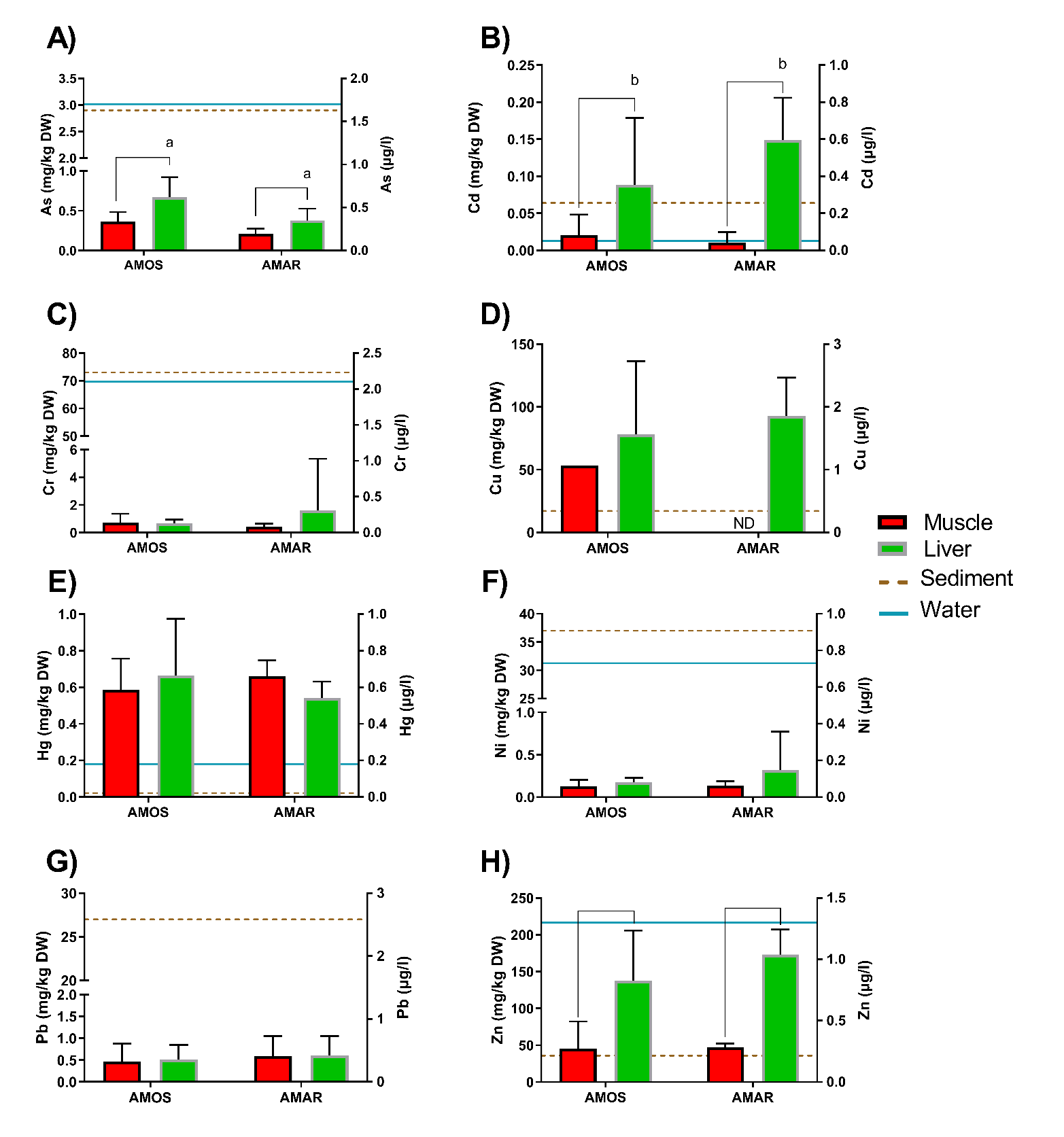


**Figure S1:** Mean concentrations (mg/kg DW) of As (A), Cd (B), Cr (C), Cu (D), Hg (E), Ni (F), Pb (G), and Zn (H) with standard deviation of the mean in the muscle and liver tissue of two eel species (*Anguilla mossambica* (n = 7), and *Anguilla marmorata* (n = 12)) collected from the Incomati River, Mozambique. Mean element concentrations in the sediment (mg/kg DW) were indicated on the left y-axis with a brown dashed line, while dissolved element concentrations in water (µg/l) were indicated on the right y-axis with a blue solid line. Dissolved concentrations of Cu and Pb in the water were below the detection limit. Brackets indicate significant differences between muscle and liver in one species, while common alphabetic superscripts indicate significant differences between species. Concentrations of Cu in muscle tissue of *A. mossambica* were only detected in one individual, while in *A. marmorata* all the Cu concentrations in the muscle tissue were below the detection limit of 0.11 mg/kg DW.
